# Effects of Water and Nitrogen Regulation on Soil Environment and Crop Growth in a *Lycium barbarum*||Alfalfa System

**DOI:** 10.3390/plants13233348

**Published:** 2024-11-29

**Authors:** Yanlin Ma, Wenjing Yu, Wenjing Chang, Yayu Wang, Minhua Yin, Yanxia Kang, Guangping Qi, Jinghai Wang, Yuping Zhao, Jinwen Wang

**Affiliations:** College of Water Conservancy and Hydropower Engineering, Gansu Agricultural University, Lanzhou 730070, China; mayl@gsau.edu.cn (Y.M.); 14760650228@163.com (W.Y.); 18893913103@163.com (W.C.); wangyy@gsau.edu.cn (Y.W.); qigp@gsau.edu.cn (G.Q.); wangjh@gsau.edu.cn (J.W.); 13830251518@163.com (Y.Z.); 17899318896@163.com (J.W.)

**Keywords:** *Lycium barbarum*||alfalfa system, water and nitrogen regulation, soil environment, model

## Abstract

The increasing scarcity of water and soil resources, combined with inefficient water and fertilizer management, poses significant challenges to agriculture in arid regions. This study aimed to determine an optimal water and nitrogen regulation model to alleviate water shortages and improve agricultural productivity and quality. In this study, a two-year experiment was conducted to investigate the effects of varying irrigation and nitrogen levels on the soil environment and crop growth in a *Lycium barbarum*||alfalfa system (LB||AS). The experiment involved four moisture gradients and four nitrogen application levels (using urea as the nitrogen source). The results indicated that soil moisture decreased during crop development, followed by a slow increase, with significant variation across soil depths. Soil temperature peaked during the fruiting stage of *Lycium barbarum* in July, decreasing significantly with soil depth. Higher temperatures were recorded in N0 under the same irrigation level and in W3 under the same nitrogen level. Soil organic carbon (SOC) levels increased by 16.24% in W3N0 and by 18.05% in W2N1, compared to W0N3. Easily oxidizable organic carbon (EOC) and soluble organic carbon (DOC) levels exhibited significant variations depending on irrigation and nitrogen treatments. Irrigation and nitrogen had a stronger individual impact on alfalfa height and stem thickness than their combined effects. Water and nitrogen regulation significantly influenced *Lycium barbarum* yield, its 100-fruit weight, and economic efficiency (*p* < 0.05). The W0N2 treatment produced the highest yield (3238 kg·ha^−1^), exceeding other treatments by up to 29.52%. In conclusion, the optimal water–nitrogen regulation model for the LB||AS system is full irrigation (75–85% *θ_fc_*) with a nitrogen application rate of 300 kg·ha^−1^. These findings offer critical insights for improving water and nitrogen management strategies in arid regions.

## 1. Introduction

Water and soil resource shortage problems are increasing and have become a major factor restricting economic and social development and ecological environment security, coupled with irrational irrigation and fertilization, resulting in ecological environment damage [1,2]. Exploring a reasonable irrigation and fertilizer application mode is the key to alleviating water shortages, improving the soil environment, and enhancing farmland productivity at the present time. Forest-grass intercropping is an important production method for the sustainable development of agriculture in the dry zones of Northwest China, which not only improves land-use efficiency but also addresses ecological environmental problems such as soil acidification and sloughing [3]. *Lycium barbarum* has the advantages of strong drought resistance, nutrient richness, and high economic value, and it is the main economic tree species in Northwest China used to improve the ecological environment and promote farmers’ income [4]. However, the monoculture of *Lycium barbarum* faces several challenges, including sparse vegetation cover and high demands for water and fertilizers [5]. Alfalfa, often referred to as the “king of forage,” is renowned for its high yield, excellent nutritional quality, and ability to improve soil conditions and prevent wind and sand erosion [6]. The intercropping of *Lycium barbarum* with alfalfa offers a promising approach to overcoming the limitations of monoculture systems, with the potential to improve soil health, enhance land productivity, and increase biodiversity.

Water and nitrogen are key factors affecting plant growth and development [7]. Water, as an indispensable medium for plants, participates in physiological processes such as photosynthesis, nutrient transport, and substance synthesis, and plays a decisive role in plant growth and development and environmental adaptation [8]. Nitrogen is a basic nutrient element that is essential for plant growth, which not only plays an important role in substance metabolism but also significantly affects plant growth, yield, and quality [9]. In recent years, significant progress has been made in the study of water and nitrogen regulation, revealing the interaction mechanism between water and nitrogen in plants and exploring ways to enhance crop productivity and resource use efficiency through water and nitrogen regulation. Studies have shown that water supply directly affects crop nitrogen uptake, transport, and conversion, while nitrogen supply alters the plant’s water use efficiency [10,11]. Under suitable water conditions, an adequate nitrogen supply promotes root growth and enhances water uptake capacity, thereby increasing photosynthetic efficiency and production [12,13]. In contrast, the over-application of nitrogen fertilizer under water-stress situations may lead to the dysregulation of nitrogen metabolism and reduce water use efficiency, which, in turn, inhibits plant growth and development [14]. Important progress has also been made in the study of water and nitrogen regulation for enhancing crop tolerance. By regulating water and nitrogen supplies, plants are able to enhance their adaptability to adverse conditions such as drought and salinity. For example, under drought stress, the moderate reduction of nitrogen fertilizer application can reduce the transpiration rate and water loss [13,15]; under saline and alkaline stress, reasonable nitrogen management can promote the accumulation of osmoregulatory substances in the plant body and enhance the osmoregulation capacity of the cells, thus improving the plant’s salt tolerance [16,17]. In addition, research into water and nitrogen regulation has been applied to agricultural practices, with the aim of optimizing irrigation and fertilization strategies to improve crop yields and resource use efficiency. Studies have shown that through careful management, such as combining soil moisture monitoring and precise nitrogen fertilizer application techniques, nitrogen fertilizer loss and water wastage can be effectively reduced, resulting in increased yields and cost savings [18,19]. However, the existing research mainly focuses on annual crops; there is a lack of research on water and nitrogen regulation in perennial crops, especially integrated research on the soil environment, crop production, and the economic benefits of intercropping systems.

Based on this lack, the present study was conducted to investigate the effects of water and nitrogen regulation on the soil environment and crop production in the *Lycium barbarum*||alfalfa system (LB||AS). The primary objectives are: (1) to reveal the mechanism of the effects of different water and nitrogen supplies on the soil environment and crop production in the LB||AS, and (2) to propose a suitable water and nitrogen regulation model for the LB||AS.

## 2. Materials and Methods

### 2.1. Experimental Area Overview

**Figure 1:**
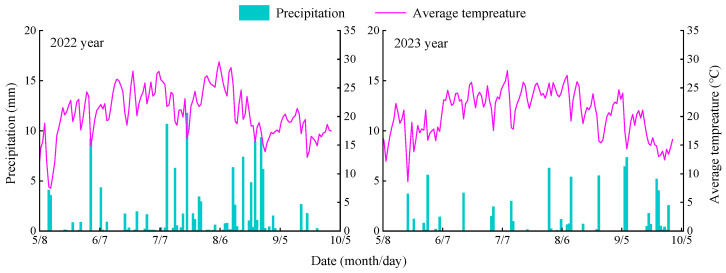
Temperature conditions.

The experiments were conducted from April 2022 to September 2023 at the Irrigation Experiment Station of the Jingtai Chuan Electricity Lift Irrigation Water Resources Utilization Centre in Gansu Province, China (latitude 37°21′55′′ N, longitude 104°08′43′′ E), at an altitude of 1562 m. This region experiences a temperate arid continental climate. The multi-year averages for sunshine hours, frost-free days, solar radiation, temperature, precipitation, and evapotranspiration are 2652 h, 191 days, 6.18 × 10^5^ J-cm^−2^, 8.6 °C, 201.6 mm, and 2761 mm, respectively. The soil in the test area consisted of 71.8% sand (2–0.02 mm), 17.4% silt (0.02–0.002 mm), and 10.8% clay (<0.002 mm), and was classified as sandy loam according to the United States Department of Agriculture (USDA) soil classification standards. Details on the physical and chemical properties of the soil are provided in Table 1. Precipitation during the experimental period was 147.29 mm in 2022 and 75.61 mm in 2023, while the average daily air temperatures were 19.07 °C and 20.58 °C, respectively. These climatic data are illustrated in Figure 1.

### 2.2. Experimental Design

**Figure 2:**
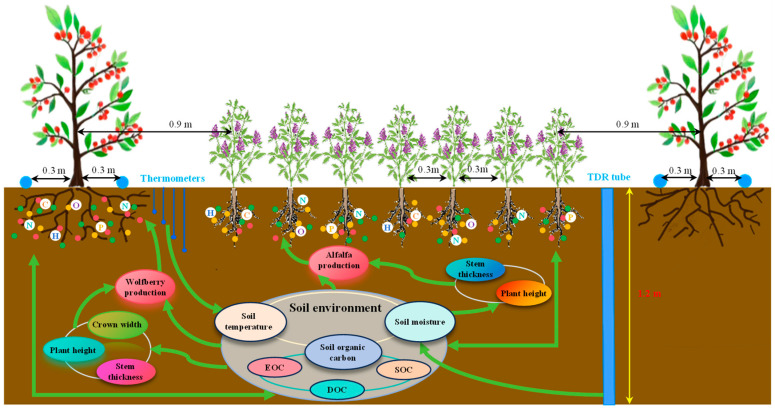
Experimental setup.

The experimental design was based on the local irrigation and fertilization systems and the team’s existing research foundation. The experiment involved four moisture gradients (W0: 75–85% *θ_fc_*, W1: 65–75% *θ_fc_*, W2: 55–65% *θ_fc_*, and W3: 45–55% *θ_fc_*) and four nitrogen application levels (N0: 0 kg·ha^−1^, N1: 150 kg·ha^−1^, N2: 300 kg·ha^−1^, and N3: 450 kg·ha^−1^), resulting in a total of 16 treatments (Table 2). Each plot measured 76.5 m^2^ (10.2 m × 7.5 m), and the experiment was replicated three times. The *Lycium barbarum* and alfalfa were established manually in April 2021. Nitrogen fertilizer was applied at a 6:2:2 ratio during the nutritive growth period (late May), flowering period (early June), and summer fruiting period (early July), respectively; the phosphorus fertilizer used was calcium superphosphate (Ca(H_2_PO_4_)_2_, with a P_2_O_5_ content of 12%) and potassium chloride (KCI, with K_2_O content of 60%), and the amount of fertilizer applied was 130 kg·hm^−2^, which was all applied at one time during the budding period of *Lycium barbarum* every year. The variety of wolfberry is “Ning Qiji 5”; it was planted in early April 2021, with a spacing of 1.5 m × 3 m, and with each plot planted in 4 rows, with 5 trees per row, comprising 20 trees in total. The alfalfa variety was “Longdong alfalfa”, sown in strips, the seeding rate was 13 kg·hm^−2^, the distance from the *Lycium barbarum* tree was 0.9 m, and the spacing between rows was 0.3 m. The specific layout of the experiment is shown in Figure 2. The test used a drip irrigation water–fertilizer integration system, with each plot having an irrigation pipe, along with the installation of valves and water meters (precision 0.0001 m^3^) to control the amount of irrigation water. The flow rate of the drip irrigation belt was 2.0 L h^−1^, laid 15 cm from the wolfberry trees. The *Lycium barbarum* trees were pruned at the beginning of April every year. Other field management and pest control practices were consistent with the local farmers’ production habits.

### 2.3. Measurement Indicators and Methodology

#### 2.3.1. Soil Water Content Testing

The soil’s water content was monitored to assess whether the soil moisture in each test plot met the irrigation threshold and to calculate the amount of irrigation water required. A 1.2 m long time-domain refractometry (TDR) probe was placed 0.9 m away from the *Lycium barbarum* trees in the plots. A PI-CO-BT TDR instrument (IMKO, Germany) was used to measure the soil’s volumetric water content in the 0–120 cm soil layer at 20 cm intervals every 3–5 days. Additional measurements were taken before and after irrigation and precipitation events. Periodic calibration was performed using the gravimetric method.

#### 2.3.2. Soil Temperature Testing

During the vegetative growth stage, full bloom stage, peak fruiting stage, and autumn fruiting stage of *Lycium barbarum*, soil temperature (5–25 cm depth) was measured every 2 h between 8:00 and 18:00 on typical sunny days, using a ground thermometer (HY-1, Hengshui City Innovative Instrumentation Co., Ltd., Hengshui, China). Soil temperature data were collected on two consecutive days for each reproductive period to ensure accuracy.

#### 2.3.3. Soil Organic Carbon Content and Fraction Testing

Soil samples were collected from four depths (0–10 cm, 10–20 cm, 20–30 cm, and 30–40 cm) during the vegetative growth stage, full bloom stage, peak fruiting stage, and autumn fruiting stage of *Lycium barbarum*. The soil samples were air-dried and sieved through a 0.25 mm mesh. Soil organic carbon (SOC) was determined using a soil organic carbon analyzer (LH-SOC350, Beijing Lianhua Yongxing Science and Technology Development Co., Ltd., Beijing, China). The readily oxidizable organic carbon (ROC) and dissolved organic carbon (DOC) fractions were measured with a spectrophotometer (T6 New Century, Beijing Pudian General Instrument Co., Ltd., Beijing, China).

#### 2.3.4. Growth and Yield Testing of *Lycium barbarum*

The plant height of *Lycium barbarum* was measured using a tape measure (from the plant base to the apex), stem thickness was recorded with a Vernier caliper (5 cm above the ground), and crown width was determined as the average of the north–south and east–west widths. In each plot, three randomly selected *Lycium barbarum* plants were monitored. Starting on August 25, fruits were harvested and dried every 7 days. The cumulative dry fruit weight from each harvest represented the total fruit yield for the year.

#### 2.3.5. Growth and Yield Tests of Alfalfa

Alfalfa height and stem thickness were measured using the same methods as for *Lycium barbarum*. During the early flowering stage of each alfalfa crop, a 1 m × 1 m sample area was randomly selected from each plot and mown, leaving 5 cm of stubble. The harvested sample was weighed to calculate the fresh weight, then dried at 105 °C for 30 min, followed by 48 h at 75 °C. The dried samples were weighed again to determine the dry weight.

#### 2.3.6. Economic Benefits

Net economic benefit was calculated as the difference between total revenue and total costs, while the benefit-to-cost ratio was determined as the ratio of total revenue to total costs. Total revenue was derived from the production of dried *Lycium barbarum* fruit and alfalfa hay, multiplied by the market prices. Total costs included the expenses for fertilizers, irrigation, management, and labor (including tillage, irrigation, pruning, pest and weed control, and fruit harvesting).

### 2.4. Data Analysis

Microsoft Excel 2019 was used to organize the data and Origin 2021 was used for plotting the data. Significance analyses were performed using the multiple comparisons (least significant difference test, or LSD) method of a one-way analysis of variance (ANOVA) (analyzed using SPSS software at a significance level of *p* < 0.05, ver. 20.0, IBM Analytics, New York, NY, USA).

## 3. Results and Analyses

### 3.1. Effects of Water and Nitrogen Regulation on the Soil Environment of LB||AS

#### 3.1.1. Soil Water Content

**Figure 3:**
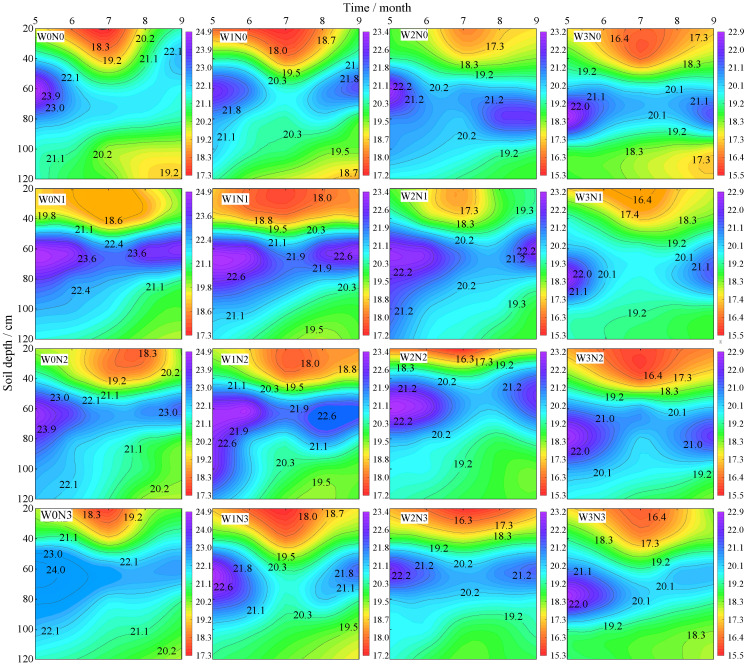
Effects of water and nitrogen regulation on soil water content.

The soil water content under varying water and nitrogen conditions exhibited a pattern of initial decrease, followed by an increase over time, reaching its lowest point in July. Additionally, it showed an increase and then a decrease with increasing soil depth, with a notable accumulation of moisture observed in the 40–80 cm soil layer (Figure 3). At the same irrigation level, soil water content was ranked as N3 > N2 > N1 > N0, with N3, N2, and N1 increasing by an average of 1.34–2.04%, 0.57–2.49%, and 0.52–1.57%, respectively, compared to N0. At identical nitrogen application levels, soil water content followed the order of W0 > W1 > W2 > W3, with W0 exhibiting increases of 3.49–6.14%, 5.9–11.58%, and 10.98–12.46% over W1, W2, and W3, respectively.

#### 3.1.2. Soil Temperature

**Figure 4:**
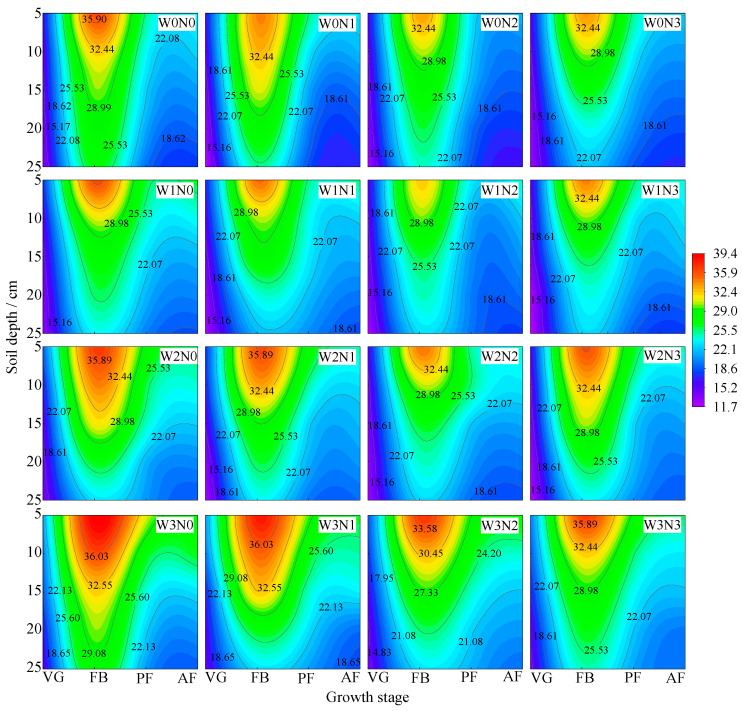
Effects of water and nitrogen regulation on soil temperature. Note: VG stands for vegetative growth stage; FB is the full bloom stage, PF is the peak fruiting stage, and AF is the autumn fruiting stage.

Soil temperature varied with the growth stages, initially increasing and then decreasing, peaking in July (the full fruit stage). The temperature at depths of 10 cm, 15 cm, 20 cm, and 25 cm decreased by 1.28–5.32 °C, 3.2–8.71 °C, 5.96–11.23 °C, and 7.44–15.21 °C, respectively, compared to that at 5 cm (Figure 4). At the same irrigation level, the soil temperature showed the order of N0 > N1 > N3 > N2, and the average increase in N0 was 7.60–15.37 °C, 4.45–9.52 °C and 2.04–5.97 °C compared with N1, N2 and N3, respectively. At the same nitrogen application level, the soil temperature was W3 > W2 > W1 > W0, and W3 was 7.60–15.37 °C, 4.45–9.52 °C and 2.04–5.97 °C higher on average than W0, W1 and W2, respectively.

#### 3.1.3. Soil Carbon Fractions

**Figure 5:**
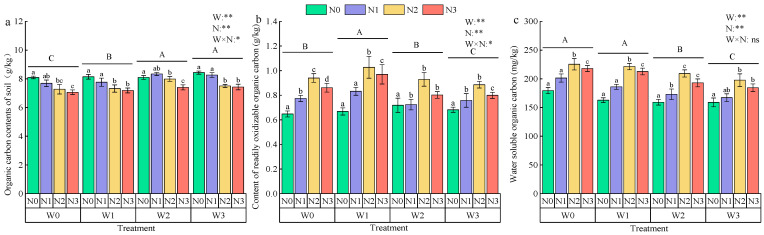
Effects of water and nitrogen regulation on soil carbon fractions. Note: Different lowercase letters indicate that there is a significant difference between different nitrogen application rates under the same irrigation amount, and different uppercase letters indicate that there is a significant difference between different irrigation amounts (*p* < 0.05). In the analysis of variance, * and ** indicated that the correlation reached a significant level (*p* < 0.05) and a very significant level (*p* < 0.01), respectively, and ns indicated that the correlation was not significant (*p* > 0.05).

Nitrogen application significantly influenced the soil organic carbon (SOC) content, although the interaction between irrigation and nitrogen did not have a significant effect (Figure 5a). At constant irrigation levels, the SOC content decreased with increasing nitrogen application. Notably, the SOC content at W0 was significantly lower than that at W2 and W3 for the same nitrogen application level. The SOC content generally increased with greater irrigation deficits, with W3N0 exhibiting the highest SOC content (8.44 g kg^−1^), surpassing W2N1 and W3N1 by 16.24% and 18.05%, respectively.

Both nitrogen application and the interaction with irrigation significantly affected the soil’s easily oxidizable carbon (EOC) content (Figure 5b). The EOC content was significantly higher in N1, N2, and N3 compared to N0 at the same irrigation level. At constant nitrogen levels, the EOC content increased initially and then decreased with increasing water deficit, showing significant increases of 8.49%, 10.19%, and 11.89% in W1 compared to W0, W2, and W3, respectively. Among all treatments, W1N2 exhibited the greatest EOC content (1.03 g kg^−1^).

Nitrogen application also significantly influenced the dissolved organic carbon (DOC) content, with no significant interaction effect (Figure 5c). At the same irrigation level, the DOC content increased initially and then decreased with higher nitrogen applications, with N1, N2, and N3 increasing by 16.81%, 48.38%, and 36.96%, respectively, compared to N0. At identical nitrogen application levels, the DOC content rose with increased irrigation water, with W0 showing average increases of 5.27%, 12.19%, and 16.34% compared to W1, W2, and W3, respectively. W0N2 had the highest DOC content (225.44 mg kg^−1^), which was 38.44% and 41.58% lower than W1N0 and W2N0, respectively.

### 3.2. Effects of Water and Nitrogen Regulation on Crop Growth in LB||AS

#### 3.2.1. Growth of *Lycium barbarum*

**Figure 6:**
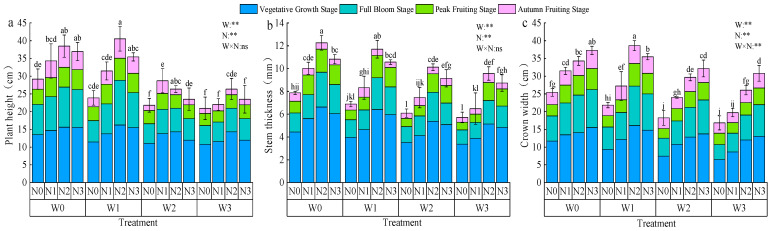
Effects of water and nitrogen regulation on the growth of *Lycium barbarum*. Note: Different lowercase letters indicate that there are significant differences between different nitrogen application rates under the same irrigation amount (*p* < 0.05). In the analysis of variance, ** indicated that the correlation reached a very significant level (*p* < 0.01), and ns indicated that the correlation was not significant (*p* > 0.05).

During the nutritive growth and blooming periods, the average height of *Lycium barbarum* increased sharply by 13.8 cm and 8.09 cm, respectively. In the blooming and autumn fruiting periods, height-related growth slowed, averaging 4.67 cm and 3.31 cm (Figure 6a). At the same irrigation level, height-related growth exhibited a trend of first increasing and then decreasing with higher nitrogen application, peaking at N1. At identical nitrogen levels (excluding N2), height-related growth decreased with an increasing water deficit, with W0 showing increases of 5.22% to 19.2%, 19.51% to 33.79%, and 39.33% to 57.16% compared to W1, W2, and W3, respectively. The highest height was recorded for the W1N2 treatment.

The average increase in stem thickness of *Lycium barbarum* was 55.70% and 22.61% during the nutritive growth and bloom periods, respectively, and 15.1% and 6.6% during the fruiting and autumn fruiting periods, respectively (Figure 6b). Stem thickness growth at the same irrigation level followed a similar trend to height growth, while at the same nitrogen application level, the stem thickness decreased with increasing water deficit, with W0 showing increases of 2.44% to 20.5%, 18.41% to 34.39%, and 23.97% to 54.64% compared to W1, W2, and W3, respectively. The highest stem thickness at full growth was recorded in W0N2 (12.26 mm), which was 23.65% and 12.52% higher than for W0N0 and W3N0, respectively.

Crown growth exhibited a similar pattern, with faster increases during the nutritive growth and full flowering stages and slower growth during the full fruiting and autumn fruiting stages (Figure 6c). At the same nitrogen application level (excluding N2), crown growth increased with more irrigation. At the same irrigation level, crown growth (excluding W1) also increased with higher nitrogen application. The largest crown growth was recorded in W1N2 (38.62 cm), which was 43.48% and 56.44% higher than in W1N0 and W3N0, respectively.

#### 3.2.2. Alfalfa Growth

**Figure 7:**
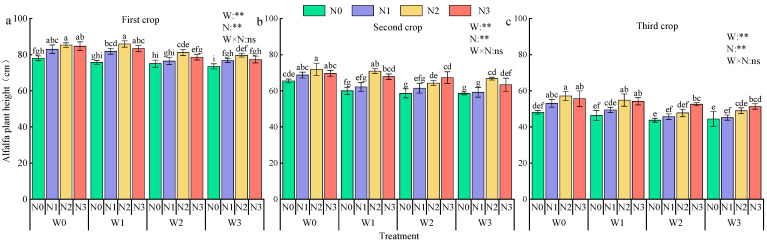
Effects of water and nitrogen regulation on the height of alfalfa plants. Note: Different lowercase letters indicate that there are significant differences between different nitrogen application rates under the same irrigation amount (*p* < 0.05). In the analysis of variance, ** indicated that the correlation reached a very significant level (*p* < 0.01), and ns indicated that the correlation was not significant (*p* > 0.05).

Overall, alfalfa plant height followed the trend of first crop > second crop > third crop, significantly influenced by nitrogen application (Figure 7). Irrigation enhanced alfalfa height by 1.21–5.68%, 6.31–12.78%, and 7.69–12.71% in the first, second, and third crops, respectively. Nitrogen application also increased alfalfa height by 3.66–5.88%, 9.85–14.27%, and 7.2–17.03% in the first, second, and third crops, respectively (*p* < 0.05). At the same irrigation rate, the height increased by an average of 6.55%, 2.12%, and 15.91% in N2 compared to N0, N1, and N3. Maximum plant height was observed under W1N2, W0N2, and W0N3 treatments, respectively.

**Figure 8:**
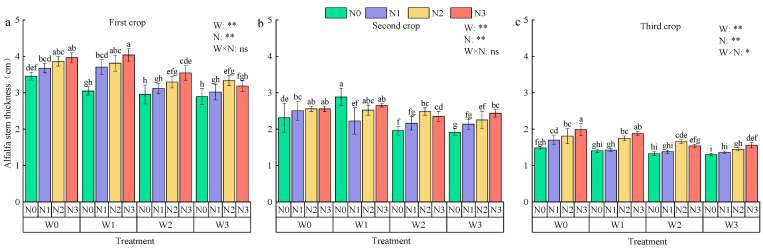
Effects of water and nitrogen regulation on alfalfa stem thickness. Note: Different lowercase letters indicate that there are significant differences between different nitrogen application rates under the same irrigation amount (*p* < 0.05). In the analysis of variance, * and ** indicated that the correlation reached a significant level (*p* < 0.05) and a very significant level (*p* < 0.01), respectively, and ns indicated that the correlation was not significant (*p* > 0.05).

Both irrigation and nitrogen application significantly influenced alfalfa stem thickness, with the interaction being significant only for the third crop, which showed reductions of 31.60% and 54.43% in the second and third crops compared to the first crop (Figure 8). With constant irrigation, the order of stem thicknesses across the three crops was N3 > N2 > N1 > N0, with N0, N1, and N2 decreasing by an average of 4.02%, 12.68%, and 21.94%, respectively, compared to N3. For the same nitrogen application, stem thickness was ranked as W0 > W1 > W2 > W3, with W0 increasing by an average of 5.02%, 15.97%, and 19.96% compared to W1, W2, and W3. The highest average stem thickness across the three crops was in W0N3 (2.95 cm), while the lowest was in W3N0 (2.04 cm).

### 3.3. Effects of Water and Nitrogen Regulation on Yield and Economic Efficiency in LB||AS

Different water and nitrogen treatments significantly affected the yield and economics of LB||AS (Table 3 and Table 4). For *Lycium barbarum*, in 2022, the 100-grain weight of *Lycium barbarum* was highest under W3 irrigation, while the maximum yield was recorded under W0 irrigation when the same nitrogen application rate was used. With fixed irrigation levels, both the 100-grain weight and yield peaked under the N3 nitrogen treatment application. The W0N2 treatment produced the highest yield (2623 kg·ha^−1^), surpassing the yields under W0N1, W0N3, and W3N3 treatments by 16.3%, 7.0%, and 67.5%, respectively. In 2023, similar trends were observed: higher 100-grain weights and yields under the W0 and W1 conditions, and lower yields under the W2 and W3 conditions. The W0N2 treatment again achieved the maximum yield (3238 kg·ha^−1^), exceeding those with the W0N1, W2N2, and W3N2 treatments by 5.93%, 23.13%, and 29.52%, respectively. Across both years, the W0N2 treatment consistently resulted in the highest yield.

For alfalfa, in 2022, the highest alfalfa yield occurred under W2 irrigation, while the lowest yield was recorded under W0 irrigation when the nitrogen application rates were consistent. At constant irrigation levels, alfalfa yield was highest under N0 and lowest under N3 conditions. The W0N2 treatment achieved the maximum yield (13,628 kg·ha^−1^). In 2023, the alfalfa yield decreased significantly with increasing irrigation volumes. Nitrogen application showed an initial positive effect, with yields peaking under N2 conditions before declining. The W1N2 treatment achieved the highest yield (14,071 kg·ha^−1^), outperforming the W0N0, W0N2, W3N0, and W3N2 treatments by 24.24%, 5.36%, 40.34%, and 18.85%, respectively.

For the combined LB||AS, the total system yield in 2022 initially increased and then decreased as irrigation levels rose. Yield decreased steadily with higher nitrogen application levels. The W0N2 treatment produced the highest system yield (16,251 kg·ha^−1^), followed by W1N2. The lowest yield (9798 kg·ha^−1^) occurred under W3N0. In 2023, system yield followed a similar trend, with a peak at intermediate irrigation levels. The highest system yield (17,117 kg·ha^−1^) was observed under W1N2, followed by W0N2. The lowest yield (9970 kg·ha^−1^) occurred under W3N0. Overall, W1N2 consistently demonstrated superior performance, with W0N2 as a close second.

For economic benefits, annual costs for treatments, including labor, utilities, fertilizers, and pesticides, ranged from USD 452.9 to USD 586.7 per hectare. In 2022, the economic benefits increased with higher irrigation volumes. Compared to W1, W2, and W3, the W0 irrigation treatment resulted in average economic benefit increases of USD 400, USD 1800, and USD 4200 per hectare, respectively. Economic benefits also increased with nitrogen application, peaking at N2. The economic benefits under N2 were USD 2900, USD 1400, and USD 1000 per hectare higher than for N0, N1, and N3, respectively. In 2023, economic benefits continued to rise with increased irrigation. Compared to W1, W2, and W3, W0 treatment improved economic benefits by an average of USD 2800, USD 14,800, and USD 17,000 per hectare, respectively. Similarly, nitrogen application benefits peaked at N2, with increases of USD 19,900, USD 11,800, and USD 1300 per hectare compared to those for N0, N1, and N3, respectively.

Under the baseline local irrigation pattern, net income was only USD 708.40 per hectare. However, optimizing irrigation and fertilization patterns significantly enhanced income across all treatments over two years. The W0N2 treatment yielded the highest net incomes, reaching USD 16,000 per hectare in 2022 and USD 20,700 per hectare in 2023, thus maximizing economic returns.

## 4. Discussion

### 4.1. Effects of Water and Nitrogen Regulation on the Soil Environment in LB||AS

Effective irrigation and fertilization programs significantly enhance soil moisture conditions. In this study, it was found that the soil water content showed a trend of decreasing and then increasing with the advancement of fertility, which was especially significant under moderate (W2) and severe (W3) water deficit conditions. This was mainly due to lower temperatures in early April, which resulted in less soil evaporation, coupled with higher surface soil water contents due to winter and spring irrigation. With the gradual rise in temperature, soil evaporation increased, coupled with rapid plant growth, resulting in a decrease in soil water content, whereas, in the later stages, when the temperature decreased and was accompanied by precipitation recharge, the soil water content gradually increased. Excessive water deficit can inhibit crop growth, reduce surface vegetation coverage, and enhance soil evaporation, which is consistent with the conclusions of Chen [20]. Conversely, excessive irrigation may lead to water leakage [21]. In addition, this study found that the soil water content tended to increase and then decrease with soil depth. The maximum soil water contents of W0 and W1 occurred in the 60 cm soil layer, while the maximum water contents of W2 and W3 were recorded in the 80 cm soil layer. Therefore, moderate water control can effectively improve the vertical distribution of soil moisture.

Soil temperature is an important ago-environmental factor, and its distribution is closely related to soil moisture status, which usually shows that the higher the moisture, the lower the temperature [22]. This study showed that the soil temperature gradually decreased with increasing irrigation water, and this trend was particularly significant under moderate (W2) and heavy (W3) moisture regulation, indicating that irrigation had a cooling effect on the soil. The temperature and its variations decrease with increasing soil depth, indicating that deeper soil temperatures are more stable and less influenced by meteorological factors [23]. Moreover, Chen [24] indicated that as nitrogen application increases, soil temperature across various layers decreases. This may be due to the rapid conversion rate of nitrogen fertilizer in sandy soils, leading to poor moisture retention and rapid heat loss, thereby lowering the soil temperature.

The soil’s organic carbon pool is mainly composed of reactive organic carbon and stable organic carbon, of which reactive organic carbon is a carbon source that can be directly utilized by soil microorganisms, is susceptible to plant and microbial influences, and is soluble and unstable [25]. The present study showed that there were differences in the soil organic carbon, readily oxidizable organic carbon, and water-soluble organic carbon contents in response to the water and nitrogen environment. This may be due to differences in the utilization and transformation of different active organic carbon fractions. High irrigation reduced the soil’s water-soluble organic carbon content but increased the microbial biomass carbon content; high nitrogen fertilizer application increased the water-soluble organic carbon content, which is consistent with the findings of most scholars [26,27]. Increased irrigation and nitrogen application can help in the decomposition and transformation of soil organic matter, enhance microbial activity, and improve the microbial community structure, thus releasing more active organic carbon [28]. In addition, nitrogen application promotes the production of apoplastic substances and increases exogenous carbon inputs, which in turn further elevate the content of reactive organic carbon [29]. However, it has also been shown that high nitrogen application increases the soil’s carbon loss, especially when crop residues are not returned to the soil, leading to a reduction in below-ground biomass, which thereby reduces the source of reactive organic carbon [30]. Appropriate water and nitrogen regulation can help to increase the content of readily oxidizable organic carbon, but high levels of irrigation and nitrogen application may lead to a decrease in the content of readily oxidizable organic carbon, which is consistent with the findings of Li [31]. It was found that high levels of irrigation led to the mineralization of the soil’s easily oxidizable organic carbon, which reduced its content. In addition, increasing the amount of irrigation water would also cause water-soluble organic carbon in the surface soil to leach to the deeper soil layers [32]. The effect of nitrogen application on the soil’s organic carbon and its fractions was more significant, indicating that nitrogen fertilization has a cumulative effect on soil organic carbon.

### 4.2. A Suitable Water and Nitrogen Regulation Model for LB||AS

Plant height, stem thickness, and crown width are vital indicators of plant health and yield prediction, and optimal water and nitrogen regulation can enhance crop growth. In this study, we found that *Lycium barbarum* plant height, stem thickness, and crown spread first increased and then decreased with the increase in nitrogen application, indicating that excessive nitrogen application may inhibit growth. *Lycium barbarum* plant height, stem thickness, and crown width increased with the increase in irrigation water, but the growth rate gradually slowed down with the advancement of the fertility period [33]. *Lycium barbarum* plant height was greatest under the W1N2 treatment and stem thickness was greatest under the W0N2 treatment, while crown spread was greatest under the W0N2 treatment. No significant changes in *Lycium barbarum* plant height were observed with increasing irrigation and nitrogen application. The main reason was that the plant would preferentially use the absorbed nutrients for nutrient growth during the growth period; as the fruit gradually matured, the competition between reproductive and nutrient growth for water and fertilizer intensified, resulting in a slowdown or cessation of plant growth [34]. In the case of alfalfa, the plant height and stem thickness decreased gradually with increasing crop size. This is mainly due to the fact that multiple mowings gradually deplete soil nutrients, which, in turn, affects plant nutrient uptake [35]. However, alfalfa plant height and stem thickness were significantly enhanced with increasing nitrogen application. This was attributed to enhanced photosynthesis and plant growth with increased nitrogen supply [36]. Alfalfa plant height was at maximum under W1N2 treatment, while stem thickness was at maximum under W0N3 treatment. This suggests that water and nitrogen combinations affect the resource allocation strategy of alfalfa.

Appropriate water and nitrogen regulation can significantly improve crop water and fertilizer uptake, resulting in optimal yields and greater economic benefits [37]. The results of this study showed that the yield of *Lycium barbarum* increased with an increase in irrigation level, and, under the same irrigation level, the yield increased and then decreased with the increase in nitrogen application, and reached the maximum level under W1N2 treatment, while the yield of alfalfa increased significantly with the increase in irrigation. This was attributed to the fact that a suitable water and fertilization environment is conducive to increasing stomatal conductance and a higher photosynthetic rate in crop leaves [38]. However, excessive inputs of water and nitrogen did not further increase the crop yield but, rather, inhibited it [39]. In this study, it was found that the yield of LB||AS first increased and then decreased with the increase in nitrogen application, and gradually decreased with the increase in water irrigation. This is mainly because excessive nitrogen fertilizer can lead to an imbalance in plant nitrogen metabolism, affecting root growth and nutrient uptake, while excessive irrigation can cause a decrease in soil porosity, leading to root hypoxia and affecting normal plant metabolism.

When the water content is insufficient, a moderate increase in nitrogen fertilizer application can alleviate the inhibition of nutrient uptake by water deficit and promote plant growth [40]. In the absence of water stress, excessive nitrogen application will reduce the solute potential of the crop’s inter-root soil to a certain extent, resulting in lower water potential, thereby hindering nutrient transport and affecting crop uptake. A moderate application of nitrogen helps to improve crop drought tolerance, thus promoting nutrient uptake [15]. A moderate reduction in water content has little effect on *Lycium barbarum* plant height, but over-irrigation may inhibit plant height-related growth. In contrast, adequate fertilizer supply may promote plant root growth [41]. The growth rates of plant height and stem thickness were mainly affected by the amount of irrigation water, while the growth rate of crown width was mainly related to the amount of nitrogen applied. Appropriate water and nitrogen regulation can enhance the root activity of *Lycium barbarum* and accelerate growth in terms of plant height, crown width, and stem thickness, but too high or too low a level of irrigation and nitrogen application may inhibit the absorption and utilization of soil water and fertilizer by the root system, which, in turn, leads to the slow growth of the plant; this finding is similar to the results of the study conducted by Zhang [42]. It was also found in this study that the economic efficiency of the intercropping system gradually increased with an increase in irrigation level. According to the original local irrigation pattern, the net income per hectare was only USD 708.40, which was lower than the irrigation fertilization pattern set in this study to varying degrees, with the W0N2 treatment achieving a net income per hectare of USD 20,700, which maximized the yield per unit of production and, thus, achieved the highest economic efficiency. The economic efficiency of LB||AS increased and then decreased with increasing nitrogen application, reaching a maximum under N2 conditions. This is because, within a certain range, the application of N fertilizer can significantly increase the yield and, thus, the economic benefit; however, with a further increase in N application, the efficiency of N fertilizer utilization decreases, and the additional cost of fertilizer application exceeds the increase in benefit from the yield. In conclusion, synergistic water and nitrogen regulation has a key role in increasing crop yield, optimizing water and fertilizer use efficiency, and improving crop quality. Considering these factors, the W0N2 treatment is recommended as the optimal water and nitrogen regulation model for LB||AS in the Yellow River irrigation area.

## 5. Conclusions

(1) Throughout the growth period, soil water content in the LB||AS system showed an initial decrease followed by an increase, while soil temperature rose initially and then declined, with a gradual decrease observed along with soil depth. Both irrigation and nitrogen application had significant effects on soil organic carbon (SOC), easily oxidizable carbon (EOC), and dissolved organic carbon (DOC) contents (*p* < 0.05). The W3N0 treatment had the highest SOC level, W1N2 showed the greatest EOC level, and W0N2 yielded the highest DOC level.
(2) *Lycium barbarum* plant height and stem thickness initially increased with nitrogen application but subsequently declined. Crown width increased with higher irrigation levels, with the W1N2 treatment supporting optimal growth in *Lycium barbarum*. Alfalfa plant height and stem thickness were also significantly affected by irrigation and nitrogen application. The W1N2 treatment achieved a maximum alfalfa height, with irrigation increasing the height by 1.21% to 12.78% and nitrogen increasing it by 3.66% to 17.03%. Maximum stem thickness was observed under W0N3, with irrigation reducing thickness by 4.02% to 21.94% and nitrogen reducing thickness by 5.02% to 19.96%.
(3) *Lycium barbarum* achieved the highest yields under the W0N2 treatment in both years, while alfalfa yield decreased with increasing irrigation. In the LB||AS, the W1N2 (17,117 kg·ha^−1^) and W0N2 (16,251 kg·ha^−1^) treatments delivered the highest system yields. The W0N2 treatment also achieved the highest economic efficiency, with net incomes of 1.6 × 10^4^ USD·ha^−1^ in 2022 and 2.07 × 10^4^ USD·ha^−1^ in 2023. Overall, a water and nitrogen regulation model combining adequate irrigation (75–85% *θ_fc_*) and a nitrogen application of 300 kg·ha^−1^ proved optimal for the LB||AS in the Yellow River irrigation area.

## Figures and Tables

**Table 1:** Physicochemical properties of the soil in the experimental sites.

Indicator	Content	Soil Type
pH	8.1	Sandy loam
Organic matter/(g·kg^−1^)	6.09	
Total nitrogen/(g·kg^−1^)	1.62	
Quick-acting nitrogen/(mg·kg^−1^)	74.5	
Quick-acting phosphorus/(mg·kg^−1^)	26.31	
Soil density (g·cm^−3^)	1.35	
Field water capacity (%)	24.1	

**Table 2:** Experimental design.

Treatment	Irrigation Level (% *θ_fc_*)		Nitrogen Application Level (kg·ha^−1^)
W0N0	Fully irrigated	75~85	0
W0N1			150
W0N2			300
W0N3			450
W1N0	Mild water deficit	65~75	0
W1N1			150
W1N2			300
W1N3			450
W2N0	Moderate water deficit	55~65	0
W2N1			150
W2N2			300
W2N3			450
W3N0	Severe water deficit	45~55	0
W3N1			150
W3N2			300
W3N3			450

**Table 3:** Effects of water and nitrogen regulation on yield and economic efficiency in LB||AS.

Year	Treatment	Alfalfa				*Lycium barbarum*		Economic Benefits /×10^4^ USD·ha^−1^
		First Crop/kg·ha^−1^	Second Crop/kg·ha^−1^	Third Crop/kg·ha^−1^	Total Yield/kg·ha^−1^	Hundred-Grain Weight/g	Yield/kg·ha^−1^	
2022	W0N0	5761 ± 124.3 d	2809 ± 103.36 g	1868 ± 74.49 cde	10,438 ± 204.52 fg	16.26 ± 0.75 f	1902 ± 39.33 f	1.18
	W0N1	6455 ± 128.13 c	3297 ± 87.26 cde	2189 ± 96.74 b	11,941 ± 152.18 cd	18.05 ± 1.02 cdef	2255 ± 41.17 d	1.38
	W0N2	7467 ± 170.82 a	3755 ± 83.68 a	2407 ± 122.28 a	13,628 ± 223.49 a	20.03 ± 1.61 abcd	2623 ± 82.04 a	1.60
	W0N3	6938 ± 180.29 b	3584 ± 53.1 b	2258 ± 120 ab	12,779 ± 144.78 b	19.13 ± 1.3 abcde	2451 ± 85.38 bc	1.50
	W1N0	5607 ± 154.53 d	2870 ± 56.02 g	1770 ± 122.28 def	10,247 ± 240.58 g	16.92 ± 0.28 ef	1900 ± 76.79 f	1.17
	W1N1	6511 ± 128 c	3206 ± 78.34 de	1992 ± 66.83 c	11,710 ± 185.94 d	18.55 ± 0.56 bcdef	2248 ± 36.25 d	1.37
	W1N2	6964 ± 161.75 b	3621 ± 97.2 b	2287 ± 75.36 ab	12,872 ± 249.86 b	20.83 ± 1.7 ab	2521 ± 62.07 ab	1.54
	W1N3	6661 ± 212.08 c	3326 ± 90.08 cd	2154 ± 92.42 b	12,141 ± 153.4 c	20.3 ± 1.3 abc	2340 ± 64.85 cd	1.43
	W2N0	5147 ± 91.01 e	2560 ± 35.52 h	1642 ± 144.5 f	9349 ± 167.92 h	17.57 ± 1.12 def	1873 ± 66.76 f	1.14
	W2N1	5859 ± 85.38 d	2781 ± 68.08 g	1764 ± 100.63 def	10,403 ± 79.02 g	19.78 ± 1.87 abcd	1987 ± 40.8 f	1.22
	W2N2	6649 ± 190.95 c	3365 ± 80.61 c	1918 ± 132.42 cd	11,932 ± 178.45 cd	19.74 ± 1.49 abcd	2118 ± 98.76 e	1.32
	W2N3	6410 ± 150.39 c	3180 ± 72.64 e	1725 ± 87.09 ef	11,316 ± 260.4 e	18.59 ± 1.15 bcdef	1995 ± 74.6 f	1.24
	W3N0	4532 ± 124.44 f	2414 ± 57.87 i	1427 ± 74.64 g	8373 ± 21.85 i	19.5 ± 1.79 abcd	1425 ± 63.94 i	0.89
	W3N1	5180 ± 174.91 e	2842 ± 71.57 g	1632 ± 31.2 f	9654 ± 274.76 h	21.24 ± 1.62 a	1567 ± 64.12 h	0.99
	W3N2	5802 ± 125.01 d	3232 ± 104.89 cde	1721 ± 77.51 ef	10,756 ± 130.09 f	21.05 ± 1.09 ab	1690 ± 94.01 g	1.08
	W3N3	5711 ± 204.16 d	3004 ± 73.38 f	1467 ± 66.49 g	10,182 ± 229.81 g	20.01 ± 0.56 abcd	1566 ± 68.18 h	1.00
2023	W0N0	4125 ± 243.37 ef	3729 ± 159.86 fgh	2802 ± 140.34 ef	10,656 ± 533.76 fgh	20.17 ± 1.07 e	2324 ± 152.87 cd	1.51
	W0N1	5103 ± 292.56 bc	4348 ± 255.01 abc	3608 ± 160.75 ab	13,059 ± 700 abc	27.45 ± 0.95 a	3067 ± 135.94 ab	1.67
	W0N2	5234 ± 393.79 ab	4415 ± 210.06 ab	3668 ± 214.91 ab	13,317 ± 814.79 ab	27.65 ± 0.80 a	3238 ± 97.36 a	2.07
	W0N3	4797 ± 221.35 bcd	4064 ± 152.19 cde	3204 ± 169.37 cd	12,065 ± 541.77 cd	23.52 ± 0.76 c	2539 ± 214.59 c	1.97
	W1N0	3608 ± 241.41 gh	3367 ± 249.63 i	2595 ± 192.73 fg	9570 ± 671.38	17.81 ± 0.56 g	2171 ± 189.68 de	1.41
	W1N1	5005 ± 345.42 bc	4343 ± 121.76 abc	3436 ± 121.34 bc	12,784 ± 588.28 bc	25.70 ± 0.61 b	3019 ± 108.31 b	1.62
	W1N2	5573 ± 166.76 a	4595 ± 137.07 a	3902 ± 103.6 a	14,071 ± 405.08 a	26.57 ± 0.78 ab	3046 ± 105.39 b	1.99
	W1N3	4749 ± 261.4 cd	4007 ± 130.13 def	3211 ± 316.65 cd	11,968 ± 698.59 cde	22.11 ± 0.97 d	2440 ± 96.57 c	1.94
	W2N0	3357 ± 185.34 hi	3063 ± 136.67 j	2510 ± 165.34 fg	8931 ± 483.09 ij	15.41 ± 0.56 hi	1836 ± 123.64 f	1.21
	W2N1	4246 ± 164.45 ef	3806 ± 131.02 efgh	2864 ± 185.98 def	12,510 ± 617.45 bcd	19.90 ± 0.55 e	2386 ± 94.51 cd	1.32
	W2N2	4960 ± 314.23 bc	4236 ± 135.24 bcd	3314 ± 171.43 bc	10,916 ± 494.77 efg	22.26 ± 0.57 d	2489 ± 88.66 c	1.61
	W2N3	3996 ± 108.56 fg	3605 ± 135.32 ghi	2759 ± 188.58 ef	10,361 ± 429.37 fgh	15.87 ± 0.56 h	1963 ± 182.8 ef	1.60
	W3N0	3171 ± 260.66 i	2915 ± 262.24 j	2310 ± 241.92 g	8395 ± 760.80 j	14.44 ± 0.41 i	1575 ± 184.9 g	1.06
	W3N1	4490 ± 143.95 de	3878 ± 132.48 efg	3902 ± 103.14 cde	12,456 ± 574.51 bcd	18.6 ± 0.95 fg	2274 ± 117.7 cd	1.40
	W3N2	4931 ± 156.92 bc	4209 ± 240.63 bcd	3317 ± 180.45 bc	11,418 ± 691.59 def	19.26 ± 0.39 ef	2282 ± 97.24 cd	1.52
	W3N3	3888 ± 165.73 fg	3504 ± 181.22 hi	2688 ± 246.32 efg	10,080 ± 592.26 gh	16.58 ± 0.65 h	2133 ± 193.08 de	1.54

Note: Different lowercase letters indicate that there are significant differences between different nitrogen application rates under the same irrigation amount (*p* < 0.05). Economic efficiency = total income (*Lycium barbarum*, alfalfa) − costs (labor, utilities, fertilizer, pesticides, etc.).

**Table 4:** Analysis of variance on yield and economic benefits under different water and nitrogen regulation conditions in LB||AS.

Year	Factors	Alfalfa				*Lycium barbarum*		Economic Benefits
		First Crop	Second Crop	Third Crop	Total Yield	Hundred-Grain Weight	Yield	
2022	W	**	**	**	**	**	**	**
	N	**	**	**	**	**	**	**
	W × N	ns	*	*	*	ns	**	**
2023	W	**	**	**	**	**	**	**
	N	**	**	**	**	**	**	**
	W × N	ns	ns	ns	ns	**	**	ns

Note: ns means not significant, * means significant at the significance level of *p* < 0.05, ** means significant at the significance level of *p* < 0.01.

## Data Availability

All data supporting this study are included in the article.
